# Inactivation of NF-κB and MAPKs confers the potent therapeutic effect of carboxyamidotriazole on Blau syndrome

**DOI:** 10.1186/s13075-026-03800-2

**Published:** 2026-03-26

**Authors:** Haoxin Song, Yang Li, Yuxin Wang, Mengyuan Duan, Na Wu, Yu Du, Ru Xu, Quanlin Chen, Min Shen, Fang Wei, Lei Zhu

**Affiliations:** 1 Department of Pharmacology, Institute of Basic Medical Sciences, Chinese Academy of Medical Sciences and School of Basic Medicine, Peking Union Medical College, Beijing, 100005 China; 2 Department of Rare Diseases, Peking Union Medical College Hospital (PUMCH), Chinese Academy of Medical Sciences & Peking Union Medical College; State Key Laboratory of Complex Severe and Rare Diseases, PUMCH, Beijing, 100730 China; 3 Department of Rheumatology and Clinical Immunology, PUMCH; National Clinical Research Center for Dermatologic and Immunologic Diseases (NCRC-DID), Ministry of Science & Technology; Key Laboratory of Rheumatology and Clinical Immunology, Ministry of Education, Beijing, 100730 China; 4 School of Pharmacy, Bengbu Medical University, Bengbu, 233030 China; 5 Medical Epigenetics Research Center, Chinese Academy of Medical Sciences, Beijing, 100005 China

**Keywords:** Blau syndrome (BS), Carboxyamidotriazole, Nucleotide-binding oligomerization domain containing 2 (*NOD2*), Nuclear factor-κB (NF-κB), Mitogen-activated protein kinases (MAPKs), Cytokines

## Abstract

**Background and objectives:**

Blau syndrome (BS) is a rare autosomal dominant autoinflammatory disease with limited treatment options, driven by the nucleotide-binding oligomerization domain containing 2 (*NOD2*) mutations that constitutively activate RIP2-mediated nuclear factor-κB (NF-κB) and mitogen-activated protein kinases (MAPKs) signaling. We have reported the potent anti-inflammatory activity of carboxyamidotriazole (CAI), originally developed as a non-cytotoxic anticancer drug. In this study, we investigated the therapeutic potential and possible mechanism of CAI for BS.

**Methods:**

Peripheral blood mononuclear cells (PBMCs) were isolated from patients with BS. Various BS-specific cellular models and animal models were established. Immunoblot, immunofluorescence and immunohistochemistry were utilized to examine the NOD2-mediated inflammatory signaling pathways. NF-κB activity was measured with a dual-luciferase reporter assay system, and cytokines were detected by ELISA.

**Results:**

In BS patient-derived PBMCs, CAI did not affect RIP2 activation under unstimulated conditions or following stimulation with muramyl dipeptide (MDP) plus lipopolysaccharide (LPS), but it suppressed NF-κB activation by reducing IKK phosphorylation and p65 nuclear translocation, ultimately leading to a marked attenuation of pro-inflammatory cytokines production. In cellular models, CAI inhibited MDP-induced activation of both NF-κB and MAPKs pathways in RAW264.7 cells, as well as the production of cytokines triggered by MDP alone or in combination with LPS. This anti-inflammatory activity was also confirmed in immortalized bone marrow-derived macrophage (iBMDM) and THP-1 cell models. Furthermore, CAI effectively suppressed constitutive NF-κB activity driven by two prevalent BS-associated NOD2 mutants (R334W and R334Q) in HEK293T cells. Finally, in animal models, CAI treatment effectively alleviated L18-MDP-induced systemic inflammation in a mouse BS model. In a rat model of BS-associated uveitis, CAI’s efficacy was further validated by its ability to ameliorate ocular pathological damage, inhibit the overactivation of NF-κB and MAPKs, and decrease the levels of pro-inflammatory cytokines in ocular tissues.

**Conclusions:**

Our findings suggest that CAI may have therapeutic value in BS by targeting the core pathogenic NF-κB/MAPK signaling axes and reducing cytokines production, which highlights CAI as a new potential medication for BS.

**Supplementary Information:**

The online version contains supplementary material available at 10.1186/s13075-026-03800-2.

## Introduction

Blau syndrome (BS) is a rare autosomal dominant autoinflammatory granulomatous disease classically characterized by the clinical triad of dermatitis, arthritis, and recurrent uveitis with onset below 4 years of age [1–3]. The gene responsible for BS has been identified in the nucleotide-binding oligomerization domain containing 2 (*NOD2*, also known as caspase recruitment domain-containing protein 15, *CARD15*) gene, which encodes a multi-domain protein, namely NOD2 [4]. NOD2 belongs to the NOD-like receptor (NLR) family [5] and functions mainly in the monocytes, macrophages, dendritic cells and intestinal epithelial cells, specifically in the recognition of muramyl dipeptide (MDP) component of the peptidoglycan which widely distributed among both Gram-positive and Gram-negative bacteria [6]. The engagement of MDP triggers NOD2 to activate receptor interaction protein-2 (RIP2), which in turn initiates downstream signaling pathways that lead to the activation of nuclear factor-κB (NF-κB) and mitogen-activated protein kinases (MAPKs). This cascade eventually induces inflammatory responses (e.g., production of pro-inflammatory cytokines) and antimicrobial molecules release, underscoring the pivotal role of NOD2 in the innate immune defense against infection [7]. However, specific *NOD2* mutations has been revealed to be linked to BS, with R334W and R334Q being the most commonly observed, found in 60–80% patients [8, 9]. The exact molecule mechanism remains unknown how *NOD2* mutations affect the pathogenesis of BS, but so far *NOD2* mutations in BS are thought to have “gain of function” which cause uncontrolled ligand-independent downstream NF-κB and MAPKs activation and excess production of pro-inflammatory cytokines, such as tumor necrosis factor (TNF)-α, interleukin (IL)-1β and IL-6 [10, 11].

There have been no sufficient data about optimal treatment for patients nowadays, owing to the rarity and the variations in the severity [10]. Treatment with systemic corticosteroids is empirically based, and in case of unsatisfactory response, additional treatment with immunosuppressive agents should be tried. The long-term uses of corticosteroids and immunosuppressants may cause severe side effects, as the disease often begins from childhood. And sometimes they still do not work. Recently, the biologic anti-cytokine agents, such as anti-TNF-α, anti-IL-1β and anti-IL-6, have represented promising therapeutic approaches in refractory cases based on the excessive production of cytokines in BS. However, the results are different which may be due to the different genotypes and phenotypes of BS, particularly with regard to ocular morbidity [10, 12–14]. Therefore, the observations of persistently active disease and poor prognosis in a majority of patients underline the need for development of potentially therapeutic agents targeted the BS pathogenesis revealed by genetics.

Carboxyamidotriazole (CAI), originally developed as a non-cytotoxic anticancer drug [15–17], showed its distinct anti-inflammatory activity by inhibiting NF-κB and MAPKs activation and reducing pro-inflammatory cytokines in our recent studies [18–26]. From this point of view, CAI might have a therapeutic effect on BS. Therefore, we systematically evaluated the efficacy of CAI on BS using patient-derived peripheral blood mononuclear cells (PBMCs), cellular models, and animal models, and explored its underlying mechanisms by investigating the NOD2-RIP2-NF-κB/MAPKs signaling pathway and resultant cytokine production.

## Methods

### Drugs

CAI was synthesized by the Institute of Materia Medica, Chinese Academy of Medical Sciences (Beijing, China). It was dissolved in dimethyl sulfoxide (DMSO) as a 40 mM stock for in vitro experiments and in polyethylene glycol 400 (PEG 400) at required concentration for in vivo experiments.

## Clinical samples and CAI treatment

PBMCs were obtained from five BS patients, including the four patients reported by us before [27]. The patients were referred to the Department of Rheumatology, Peking Union Medical College Hospital, treated and followed up by the co-authors. The demographic and genotypic data of the patients were summarized (Table S1). There were 3 women and 2 men. The median age was 25 years old (range, 8–36). All these patients had initial symptoms at their childhood, and the median age of disease onset was 5 years old (range, 2–6). When included into the study, all these patients were received intravenous TNF-α inhibitor (infliximab, 5 mg/kg) every 6 to 8 weeks plus methotrexate 10–15 mg every week. Meanwhile, one was given oral corticosteroids at a low dose. All patients achieved clinical remission. They were genotyped with whole exome sequencing by next-generation sequencing at the Center for Genetic Testing, Joy Orient Translational Medicine Research Centre Co., Ltd., Beijing, China. Two heterozygous variants including R334W and R334Q and R471C/H496P compound heterozygous were identified within the *NOD2* gene (NM_022162.1) (Table S1).

Venous blood samples were collected from BS subjects. PBMCs were isolated by Ficoll-density gradient separation and cultured in Dulbecco’s modified Eagle’s medium (DMEM) supplemented with 10% fetal bovine serum (FBS) and 1% penicillin and streptomycin (pen/strep). Then PBMCs were treated with CAI, either without stimulation or in the presence of 10 µg/mL muramyl dipeptide (MDP, Sigma, MO, USA), 10 ng/mL lipopolysaccharide (LPS, Sigma), or MDP combinated with LPS for 45 min (for detection of NF‑κB pathway activation) or 22 h (for detection of cytokine levels). This study was approved by Institutional Review Board of Peking Union Medical College Hospital and performed according to the Declaration of Helsinki (002-2018).

### Cell culture

RAW264.7, HEK293T and THP-1 cell lines were obtained from the Cell Resource Center, Peking Union Medical College (Beijing, China). Immortalized bone marrow-derived macrophage (iBMDM) line was kindly provided by Prof. Tao Li (Academy of Military Medical Sciences, Academy of Military Sciences, Beijing, China). RAW264.7, HEK293T and iBMDM cells were maintained in DMEM supplement with 10% FBS and 1% pen/strep, while THP-1 cells were maintained in 10% FBS and 1% pen/strep RPMI 1640 medium. All cells were cultured at 37 °C at 5% CO_2_.

### Animals

C57BL/6J mice (male, 20–25 g) and Sprague-Dawley (SD) rats (male, 470–490 g) were purchased from Beijing Huafukang Biotechnology Co., Ltd (Beijing, China). They were maintained on a 12:12 h day: night cycle and provided constant access to food and water. All animal procedures were approved by the Institutional Animal Care Use & Welfare Committee of Institute of Basic Medical Sciences, Chinese Academy of Medical Science (ACUC-A02-2022-098).

### Stimulant-induced BS cell models and CAI Treatment

BS cell models were established using our previously published protocol [28]. For RAW264.7 cells, treatments were performed with varying concentrations of CAI in the presence of 10 µg/mL MDP, 10 ng/mL LPS or a combination of MDP and LPS. And 100 µg/mL etanercept (ETN, Shanghai Celgen Biopharmaceutical Co., Ltd, Shanghai, China) and 1 µM GSK583 (MedChemExpress, NJ, USA) were used as the positive control drugs. Cells were incubated for 45 min to assess of NF‑κB and MAPKs activation or 22 h to measure cytokine levels. For iBMDMs, cells were treated with different concentrations of CAI, or the positive control drug, along with 10 µg/mL MDP for 22 h. For THP-1 cells, they were differentiated into macrophages by 50 ng/mL of phorbol 12-myristate 13-acetate (PMA, Beyotime Biotech Inc., Shanghai, China) for 2 days. Then the cells were treated with varying concentrations of CAI or the positive control drug, combined with 0.2 µg/mL MDP with a C18 fatty acid chain (L18-MDP, InvivoGen, CA, USA), for 22 h.

### Generation of *NOD2* mutants, transient transfection of HEK293T cells and NF-κB luciferase assay

The NOD2 cDNA was cloned into the pcDNA3.1-3×Flag vector (Tianyi Huiyuan Biotechnology Co., Ltd., Beijing, China), and deletion mutants of NOD2 were constructed by PCR. The entire coding region of all constructs was verified by sequencing, and the size of the encoded protein was confirmed by immunoblotting using anti-Flag antibody. HEK293T cells were plated in 24-well plates at a density of 4 × 10^5^ cells/well and cultured for 24 h prior to transfection. At 80–90% confluence, the cells were co-transfected with 250 ng of the firefly luciferase reporter plasmid pKM53 (Addgene, MA, USA), 2.5 ng of the Renilla control plasmid pGL4.74[hRluc⁄TK] (Promega, WI, USA), and either 250 ng of NOD2 expression plasmid (wild type (WT) or variant) or the corresponding empty vector (MOCK), using Lipofectamine 3000 (Invitrogen, CA, USA) according to the manufacturer’s instructions. After 24 h of transfection, cells were treated for another 24 h with varying concentrations of CAI in the presence or absence of 10 µg/mL MDP. Cells were then harvested, and NF-κB activity was measured using the dual-luciferase reporter assay system (Promega).

### Immunofluorescence staining

PBMCs were seeded onto 24-well plates with cover slips. After corresponding treatments, cells were fixed with 4% paraformaldehyde for 20 min at room temperature, and then permeabilized with 0.2% Triton X-100 for 10 min on ice. Next, cells were blocked with PBS containing 1% BSA for 1 h, and incubated with primary antibody against NF-κB p65 (Cell Signaling Technology, MA, USA) at 4 °C overnight. After washing with PBS, the slices were further incubated with Alexa Fluor 488-tagged secondary antibody contained in the immunofluorescent staining kit (Beyotime) for 30 min at 37℃ and counterstained with DAPI in this kit. The slices were then immediately visualized and images were captured from a DM4000 upright fluorescence microscope (Leica Microsystems, Wetzlar, Germany). Each group was performed in triplicate. The pixel intensities of p65 in the nuclear area were measured as the percent area of immunoreactivity using ImageJ software.

### Western blotting

Total proteins from cells were extracted using lysis buffer, separated by electrophoresis on 10% SDS-polyacrylamide gels, and then transferred onto polyvinylidene difluoride membranes. The membranes were incubated with specific primary antibodies overnight at 4 °C, followed by incubation with horseradish peroxidase (HRP)-coupled secondary antibodies. Subsequently, the targeted protein bands were visualized using ECL reagent in a chemiluminescence imaging system and the band densities were quantified using ImageJ software. β-actin served as the loading control, and the levels of phosphorylated proteins were normalized to their respective total protein levels.

### Mouse systemic inflammatory model of BS and CAI

C57BL/6 mice were randomly divided into seven groups (*n* = 12 per group): a normal control (CON) group, a BS model group, three CAI-treated groups (5, 10, and 20 mg/kg, p.o.), and two positive control groups (ETN and GSK583). The BS model group received only the vehicle (PEG 400, p.o.), while the CAI groups were administered corresponding doses of CAI (5, 10, or 20 mg/kg, p.o.) once daily for five consecutive days. The ETN group was treated with ETN (3.25 mg/kg, s.c.) on days 1 and 4, and the GSK583 group received GSK583 (40 mg/kg, p.o.) on day 5. One hour after administration on the fifth day, all mice except those in the control group were intraperitoneally injected with L18-MDP (8 mg/kg) [29], while control mice received an intraperitoneal injection of normal saline. Mice were sacrificed 4 h post-injection, and serum was collected for cytokines detection.

### Rat uveitis model of BS and CAI treatment

SD rats were randomly divided into four groups which included the CON group, uveitis model group, CAI group, and positive control tobramycin and dexamethasone eye drops (TD, Novartis, Switzerland) group, with *n* = 8 in each group. The uveitis model group and CAI group were administered p.o. with the vehicle PEG 400 and 40 mg/kg CAI, respectively, once daily for five consecutive days. The positive drug group received topical instillation of one drop of TD into the conjunctival sac three times daily for 5 days. One hour after the last administration on day 5, the BS-related uveitis model was established following our previously described protocol [29]. Briefly, the rats received an intravitreal injection of 2 µL of MDP (50 mg/mL) per eye, while the CON group was injected with 2 µL of normal saline. Twelve hours later, the eyeballs were enucleated for hematoxylin and eosin (H&E) staining, and the severity of uveitis was evaluated according to number and extent of lesions using the pathological Caspi scoring system (scored on a scale of 0 [no inflammation] to 4 [maximum severity]) [30].

### Immunohistochemical staining

After antigen retrieval by heat treatment, inactivation of endogenous peroxidase, and blocking, eyeballs sections were incubated with the corresponding primary antibody at 4 °C overnight. Next day, the sections were incubated with HRP-conjugated secondary antibody at 37 °C for 45 min. Peroxidase activity was visualized with 3,3-diaminobenzidine, and sections were counterstained with hematoxylin.

### Cytokine Analysis

The levels of TNF-α, IL-1β, IL-6 and IL-8 in cell culture supernatants and mouse serum were measured with enzyme-linked immunosorbent assay (ELISA) by using the corresponding kits (Shanghai ExCell Biology, Shanghai, China) according to the manufacturers’ instructions.

### Statistical analysis

The results are presented as the mean ± SD. Statistical significance for PBMCs experiments was analyzed using general linear model univariate method followed by LSD and SNK post-hoc tests by SPSS 20.0 software. To determine statistical significance for other experiments, one-way ANOVA was performed to analyze differences among multiple groups using GraphPad Prism 8.3.0 (GraphPad software, CA, USA). Values of *P* < 0.05 were considered statistically significant.

## Results

### Effect of CAI on RIP2-NF-κB pathway in PBMCs from BS patients

**Fig. 1:**
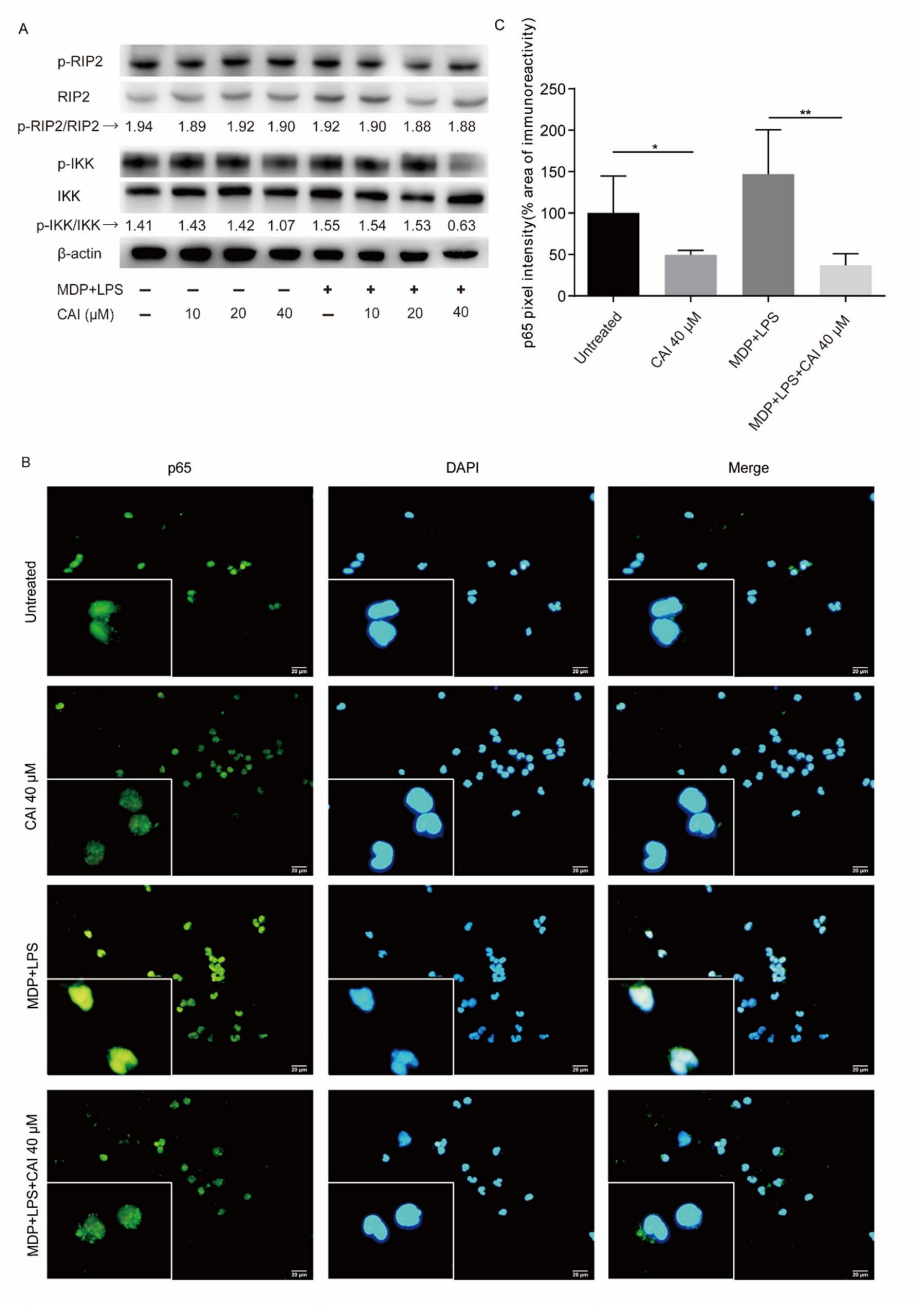
Effect of CAI on RIP2-NF-κB pathway in BS PBMCs. PBMCs from BS patients were treated with CAI in the absence or presence of MDP (10 µg/mL) and LPS (10 ng/mL) for 45 min. **A** The protein expressions of p-RIP2, RIP2, p-IKK and IKK in cell lysates were determined by Western blotting. β-actin was considered as the internal control. Immunoblot bands were analyzed for densitometry and the relative ratio of phosphorylated and total proteins was calculated. **B** Immunofluorescence was used to observe the nuclear translocation of NF-κB p65 subunit and representative images from different group were shown. **C** Quantification of nuclear NF-κB p65 fluorescence intensity in (B). The pixel intensity of the immunostained p65 in the nucleus area was quantified and the values were normalized to 100% for the expression of untreated group. The values are mean ± SD, *n* = 3 per group. *p < 0.05 and **p < 0.01

BS is associated with *NOD2* gene mutations that lead to constitutive RIP2-NF-κB signaling activation [10]. Therefore, we firstly examined the influence of CAI on this pathway in PBMCs from BS patients. As shown in Fig. 1A, the expression levels of p-RIP2 and RIP2 in BS PBMCs were not affected by CAI (10, 20 and 40 µM), but were increased following the stimulation of MDP combined with LPS. CAI (20 and 40 µM) suppressed the increased protein expressions of p-RIP2 and RIP2, but did not affect their ratio. These results indicated that CAI did not affect the phosphorylation and activation of RIP2.

NF-κB, the key downstream pathway of RIP2, was further assessed. Treatment with CAI (40 µM) markedly reduced the phosphorylation of IKK in BS PBMCs, both under unstimulated conditions and following MDP plus LPS stimulation (Fig. 1A). Given that NF-κB activation is characterized by its nuclear translocation, p65 localization was examined by immunofluorescence. CAI (40 µM) significantly decreased nuclear p65 expression in BS PBMCs under unstimulated state and also effectively impaired the enhanced nuclear translocation induced by MDP plus LPS (Fig. 1B and C). These results indicated that CAI could inhibit the overactivation of NF-κB pathway in BS patient-derived PBMCs.

### Effect of CAI on cytokines secretion in PBMCs from BS patients

PBMCs isolated from BS patients were cultured under various conditions to assess the effects of CAI on cytokine production. PBMCs from patients 1–4 were treated with a single concentration of CAI (40 µM) under unstimulated, MDP-stimulated, LPS-stimulated, or MDP + LPS co-stimulated conditions. For patient 5, to further explore the dose-dependency of CAI’s effect and given limited sample availability, PBMCs were treated with increasing concentrations of CAI (10, 20, and 40 µM) under only two conditions: unstimulated and MDP + LPS co-stimulated.

**Fig. 2:**
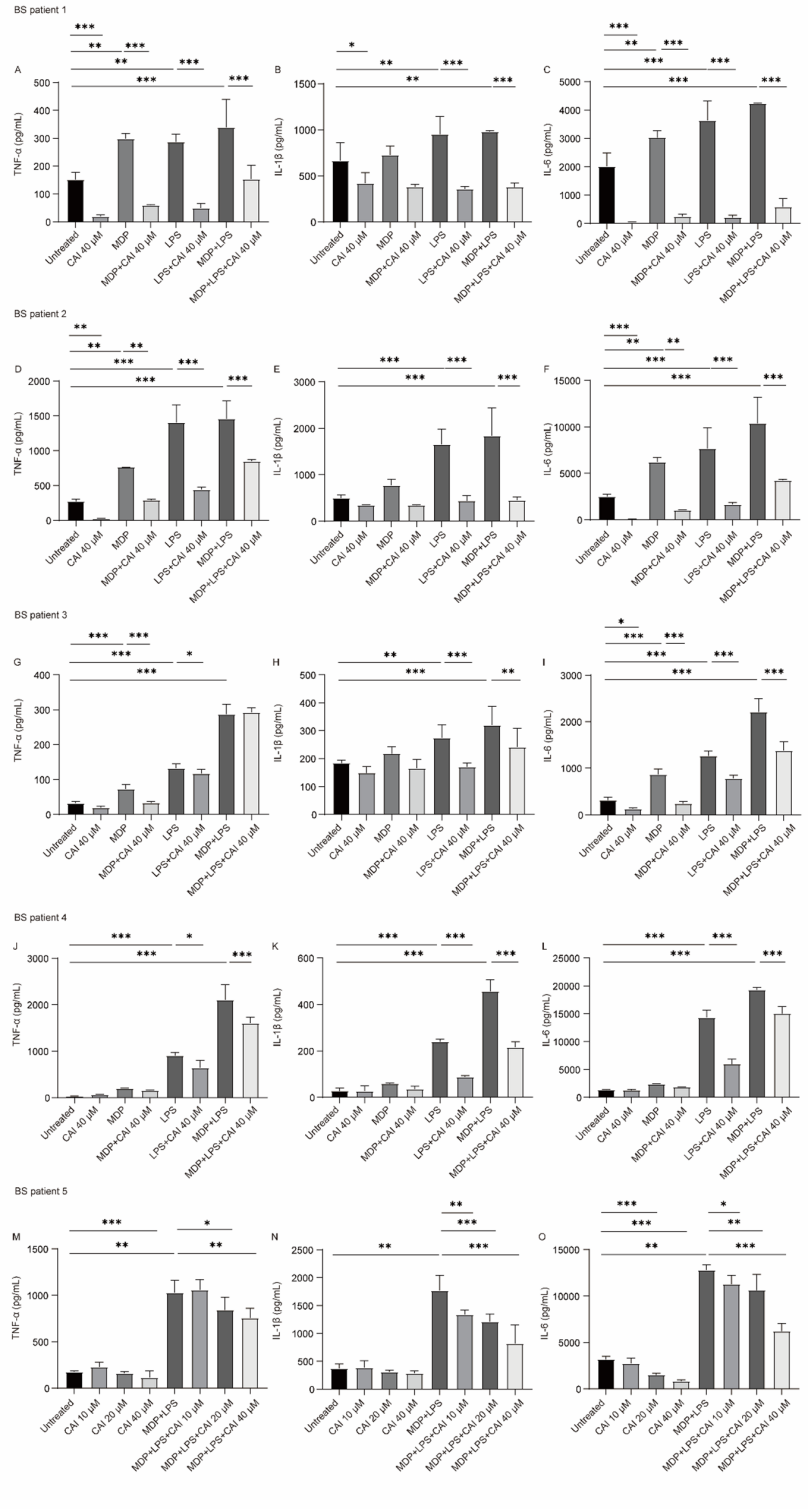
Effect of CAI on cytokines secretion from BS PBMCs. PBMCs from BS patients were treated with CAI for 22 h either without stimulation, or in the presence of MDP (10 µg/mL), LPS (10 ng/mL), or MDP combinated with LPS. The concentrations of TNF-α, IL-1β, and IL-6 in the supernatants were measured by ELISA. The values are mean ± SD, *n* = 2 ~ 5 per group. *p < 0.05, **p < 0.01 and ***p < 0.001

The results showed that basal levels of the cytokines TNF-α and IL-6 in the supernatant of PBMCs from three of the five patients (patients 1, 2 and 5) were decreased after treatment with CAI (40 µM). MDP stimulation increased TNF-α and IL-6 release in PBMCs from three of the four patients (patients 1, 2 and 3), and this increase was attenuated by CAI (40 µM); however, MDP did not elicit IL-1β secretion from any of the patients tested (patients 1–4). LPS stimulation upregulated the release of TNF-α, IL-1β, and IL-6 in all four patients (patients 1–4), and co-treatment with CAI (40 µM) suppressed this LPS-induced cytokine production. When MDP was used in combination with LPS, the synergistic enhancement of all three cytokines was observed in PBMCs from all five patients (patients 1–5). This induction was inhibited by CAI (40 µM) in all cases, with the exception that CAI did not affect MDP + LPS-induced TNF-α production in PBMCs from patient 3 (Fig. 2). Furthermore, results from PBMCs of patient 5 (Fig. 2M-O) demonstrated that the inhibitory effects of CAI on both the basal secretion of TNF-α and IL-6 and the robust production of all three cytokines induced by MDP and LPS co-stimulation were dose-dependent. Notably, when the experimental groups common to all five patients were analyzed collectively, 40 µM CAI inhibited both the basal secretion of TNF-α and IL-6 from unstimulated PBMCs and the release of TNF-α, IL-1β, and IL-6 stimulated by MDP plus LPS (Fig. S1).

### Effect of CAI on NOD2-mediated signaling and pro-inflammatory cytokines production in stimulant-induced BS cell models

**Fig. 3:**
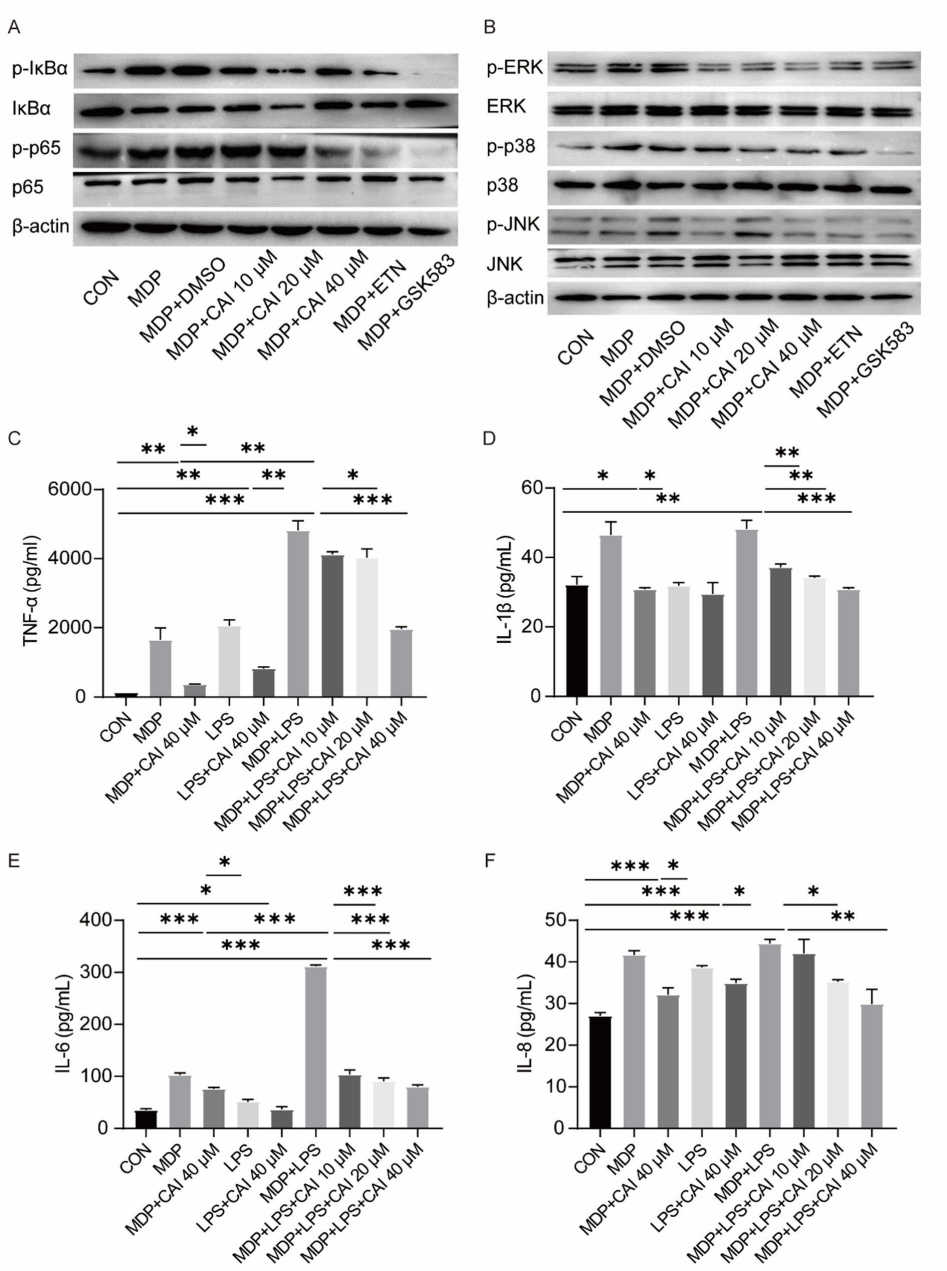
Effect of CAI on NF-κB and MAPKs activation and cytokines production in stimulant-induced RAW 264.7 cell model.** A**,** B** RAW264.7 cells were stimulated with 10 µg/mL MDP in the absence or presence of CAI for 45 min. ETN and GSK583 were used as the positive control drugs. The indicated proteins were detected by Western blotting. Results are representative of three independent experiments. **C-F** RAW264.7 cells were stimulated MDP (10 µg/mL), LPS (10 ng/mL), or a combination of MDP and LPS, in the absence or presence of CAI, for 22 h. The concentrations of TNF-α, IL-1β, IL-6 and IL-8 in the supernatants were measured by ELISA. The values are mean ± SD, *n* = 3 per group. *p < 0.05, **p < 0.01 and ***p < 0.001

After confirming CAI’s efficacy in PBMCs from BS patients, we further evaluated its effects in BS cell models. In MDP-stimulated RAW264.7 cell model, all three CAI doses and the two positive control drugs inhibited the phosphorylation and degradation of IκB, as well as p65 phosphorylation, to varying degrees. This suggested that CAI could suppress NF-κB activation in this cellular model (Fig. 3A, Fig. S2A and B). Furthermore, CAI and the positive controls significantly inhibited MDP-induced activation of the three MAPK pathways, including ERK, p38, and JNK (Fig. 3B and Fig. S2C-E). We next assessed CAI’s impact on cytokines secretion. It was found that CAI effectively reduced the secretion levels of TNF-α, IL-1β, IL-6, and IL-8 induced by MDP, LPS, or their combination (Fig. 3C-F). In addition, in the MDP-induced iBMDM model (Fig. S3) and L18-MDP-induced THP-1 cell model (Fig. S4), CAI also obviously reduced TNF-α level. These results indicated that CAI suppressed the activation of NF-κB and MAPK signaling pathways and the subsequent production of cytokines in stimulant-induced BS cell models.

### Effect of CAI on NF-κB activation in BS mutant cells

**Fig. 4:**
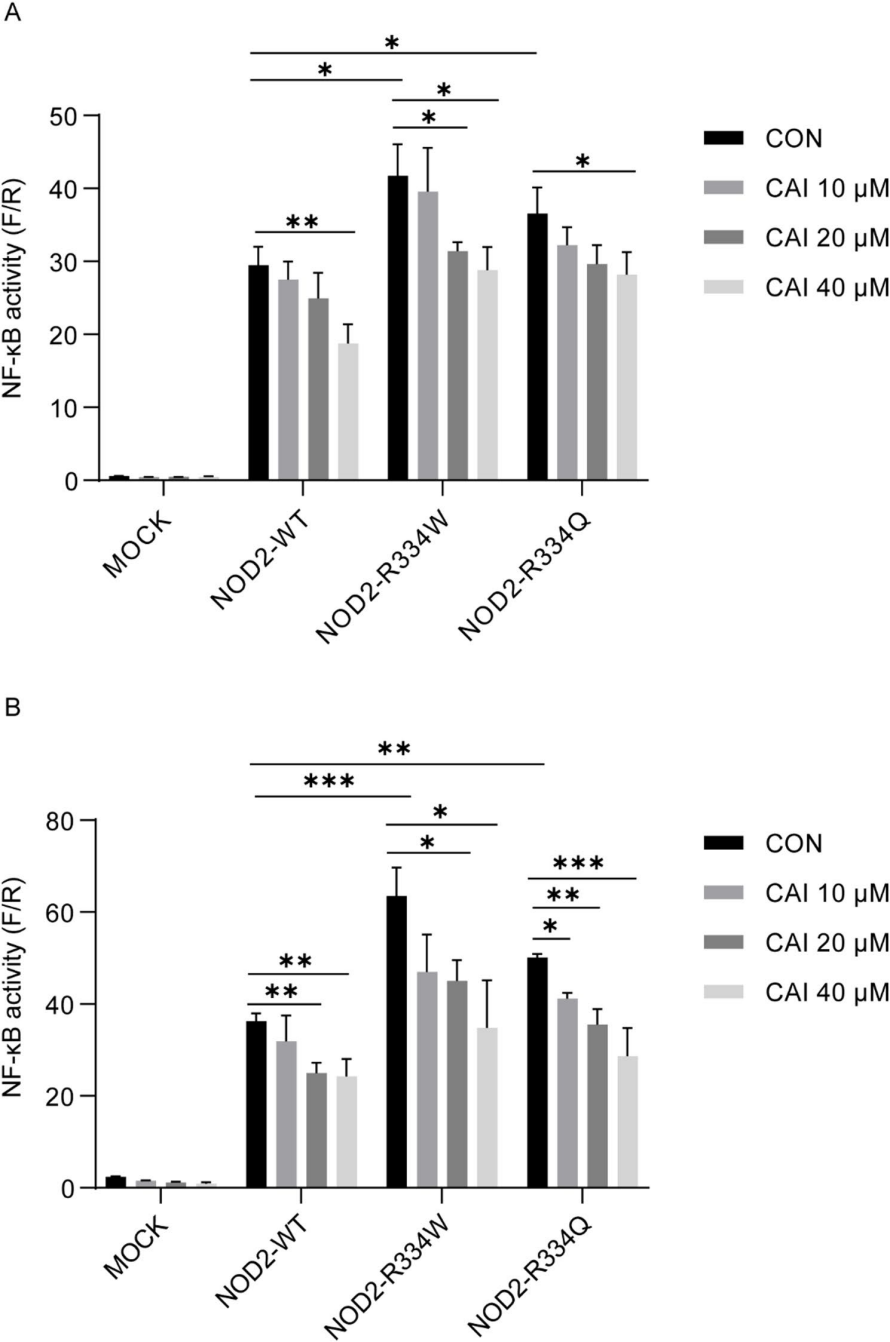
Effect of CAI on NF-κB activity in BS mutant cells. HEK293T cells were transfected with plasmids encoding NOD2-WT, NOD2-R334W, NOD2-R334Q, or MOCK. After 24 h, cells were treated for an additional 24 h with CAI in the absence **(A) **or presence **(B)** of MDP (10 µg/mL). NF-κB activity was determined using a dual-luciferase reporter assay and is presented as the ratio of firefly to Renilla luciferase activity (F/R). The values are mean ± SD, *n* = 3 per group. *p < 0.05, **p < 0.01 and ***p < 0.001

R334W and R334Q are the most common BS-associated mutations [8, 9]. Thus, we transfected these two mutations into HEK293T cells to establish the BS cell model with *NOD2* gene mutation, and evaluated the effect of CAI by detecting NF-κB activity. At equivalent expression levels, the BS-associated mutants NOD2-R334W and NOD2-R334Q exhibited enhanced basal activity compared to NOD2-WT, demonstrating their capacity for constitutive NF-κB activation. Notably, CAI treatment effectively inhibited this NF-κB activation regardless of whether cells were transfected with NOD2-WT or the disease-associated variants (Fig. 4A). Furthermore, upon MDP stimulation (Fig. 4B), CAI treatment remained effective in suppressing NF-κB activation in HEK293T cells transfected with either NOD2-WT or its variant (R334W/R334Q). These results indicated that CAI could impair the pathological NF-κB overactivation driven by BS-associated NOD2 mutations.

### Effect of CAI on BS animal models in *vivo*

**Fig. 5:**
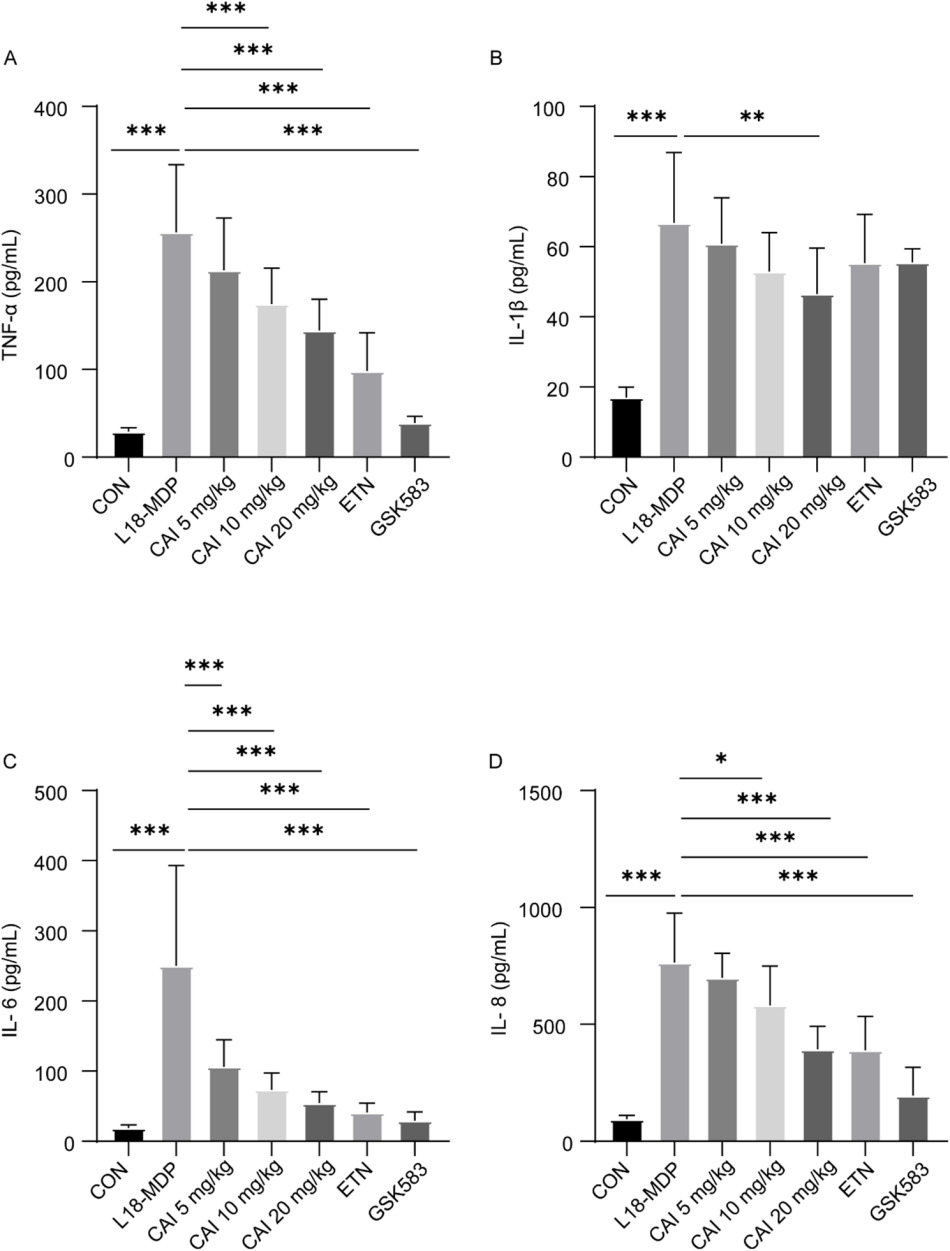
Effect of CAI on systemic inflammation in a mouse model of BS. Mice were administrated with CAI or the positive drugs (ETN and GSK583), followed by intraperitoneal injection of L18-MDP (8 mg/kg) to induce systemic inflammation. Serum levels of TNF-α **(A)**, IL-1β **(B)**, IL-6 **(C)**, and IL-8 **(D)** were measured by ELISA. The values are mean ± SD, *n* = 12 per group. *p < 0.05, **p < 0.01 and ***p < 0.001

Finally, to evaluate the efficacy of CAI in vivo, two BS animal models was established: a systemic inflammatory model in mouse by intraperitoneal injection of L18-MDP, and a uveitis model in rat via intravitreal injection of MDP. In the mouse systemic model, CAI administration dose-dependently reduced the elevated serum levels of pro-inflammatory cytokines including TNF-α, IL-1β, IL-6, and IL-8 (Fig. 5).

**Fig. 6:**
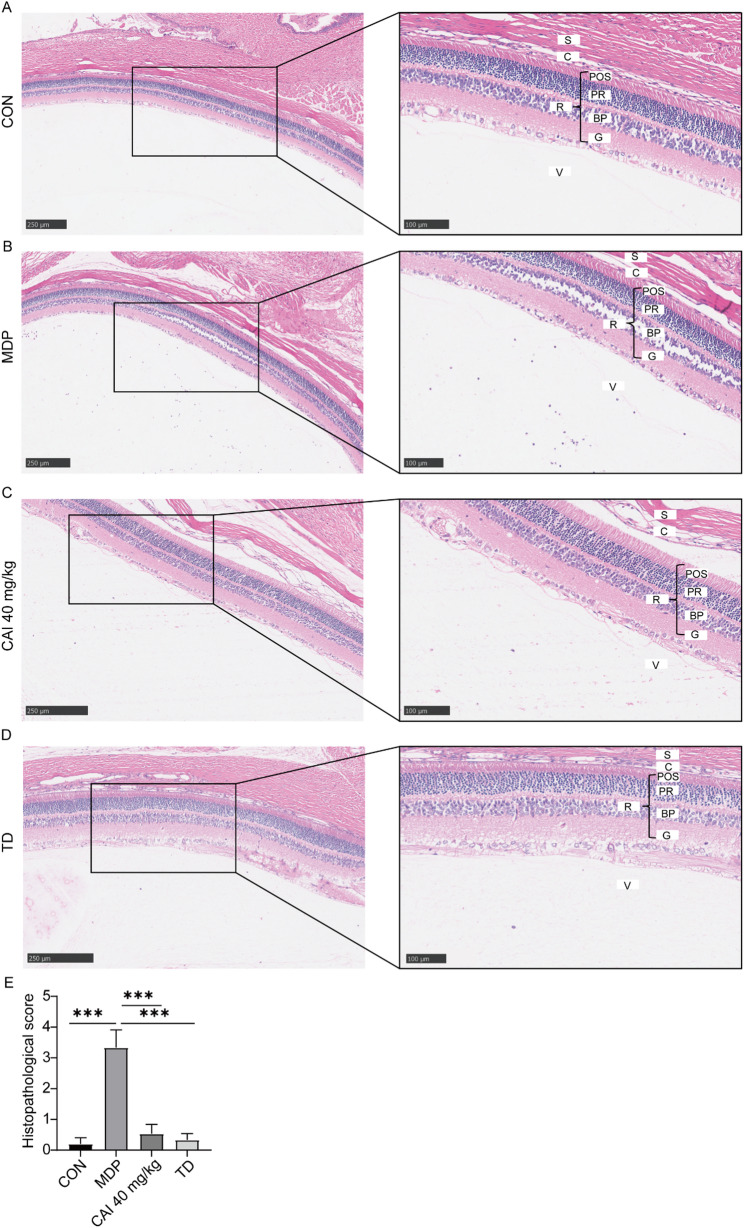
Effect of CAI on histopathological damage in a rat model of BS-associated uveitis. Rats were administrated CAI or positive drug TD, and then subjected to an intravitreal injection of intravitreal injection of 2 µL of MDP (50 mg/mL) to induce uveitis. **A-D** Representative images of H&E staining in eyeball sections from the CON (**A**), MDP-induced uveitis (**B**), CAI-treated (**C**), and TD-treated (**D**) groups. S: sclera; C: choroid; R: retina; POS: photoreceptor outer segments; PR: photoreceptor; BP: bipolar cells; G: ganglion cells; V: vitreous body. Red arrow indicates neutrophils; black arrow indicates retinal damage. **E** Histopathological changes were scored using the pathological Caspi scoring system. The values are mean ± SD, *n* = 8 per group. ***p < 0.001

**Fig. 7:**
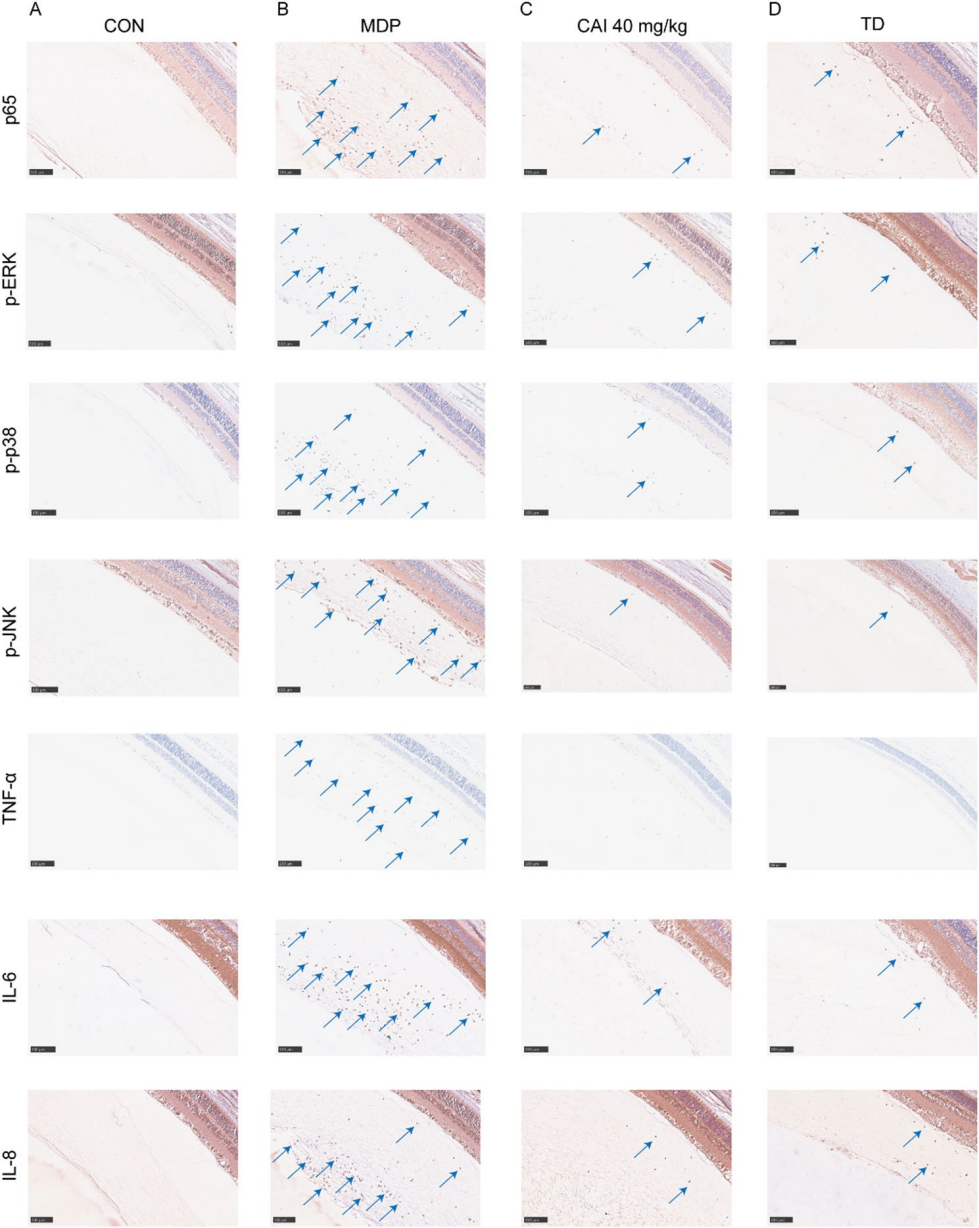
Effect of CAI on NF-κB and MAPKs activation and cytokines production in the eyeballs from BS-associated uveitis rats. Rats were administrated CAI or TD, and then subjected to an intravitreal injection of MDP to induce uveitis. Representative immunohistochemical images of p65, p-ERK, p-p38, p-JNK, TNF-α, IL-6, and IL-8 in eyeball sections from the CON **(A)**, MDP-induced uveitis **(B)**, CAI-treated **(C)**, and TD-treated **(D)** groups. Blue arrow indicates positive staining

In the rat uveitis model, MDP challenge induced severe ocular pathology, characterized by extensive inflammatory cell infiltration in the retina and vitreum, along with retinal tissue damage. However, the administration of CAI diminished diminishing both inflammatory infiltration and retinal injury, resulting in a marked reduction in the histopathological score (Fig. 6). Furthermore, immunohistochemical analysis revealed that the NF-κB p65 subunit expression in normal eyes was in a low level and predominantly cytoplasmic. However, intravitreal MDP injection triggered the pronounced increase in the expression of NF-κB p65 and promoted its nuclear translocation. CAI administration markedly attenuated the NF-κB p65 staining and suppressed its nuclear localization. Moreover, CAI potently inhibited the MDP-induced up-regulation of p-ERK, p-p38, and p-JNK, as well as pro-inflammatory cytokines TNF-α, IL-6, and IL-8, in ocular tissues (Fig. 7). These results indicated that CAI exerted significant protective effects in both mouse model of systemic inflammation and rat model of uveitis, which are relevant to BS.

## Discussion

Our previous studies reported the anti-inflammatory action of CAI, which showed good efficacy in a variety of acute and chronic animal models of inflammation, including croton oil-induced ear oedema, cotton-induced granuloma, rheumatoid arthritis, inflammatory bowel disease, Sjögren’s syndrome, and psoriasis. Mechanistic studies revealed that the anti-inflammatory activity of CAI were associated with the inactivation of NF-κB and MAPKs, as well as the downregulation of cytokines [18–26]. Therefore, we explored the effect and underlying mechanism of CAI for the treatment of BS, in which RIP2-NF-κB/MAPKs signaling aberration due to *NOD2* mutations is its main pathogenic cause.

It is widely concerned that BS is caused by gain-of-function mutations of *NOD2* [4]. NOD2 protein contains two amino-terminal caspase activation and recruitment domains (CARDs), one central nucleotide binding domain (NBD), and carboxyl-terminal leucine-rich repeats (LRRs). It exists in the cytoplasm of the resting cells in an auto-inhibitory state due to an intramolecular interaction between its LRRs and CARDs domains. Upon binding MDP ligand, NOD2 unfolds and undergoes oligomerization through the NBD domain, thus exposing the CARD-containing effector domain and inducing the recruitment of the serine-threonine kinase RIP2 via a CARD-CARD interaction. After auto-phosphorylating and activating, RIP2 initiates downstream signaling by promoting the phosphorylation of IKK and by activating the MAPKs signaling pathway. The activated IKK then phosphorylates IκB, resulting in its ubiquitination and degradation and NF-κB activation [4, 7, 31]. The mutations in BS are primarily located in the NBD domain. Since NOD2 activation involves oligomerization initiated by the NBD domain, mutations in this area are thought to reduce the threshold for spontaneous oligomerization of NOD2, leading to ligand-independent activation of the RIP2-NF-κB/MAPKs pathway and thereby driving the excessive production of pro-inflammatory cytokines [32, 33]. This concept is supported by the findings that the cells expressing BS-associated *NOD2* mutations demonstrated MDP-independent constitutive activation of NF-κB [11, 32, 33], and biologic agents targeting pro-inflammatory cytokines have been explored as a therapeutic strategy for BS [10, 12–14, 27].

In this study, we started by analyzing the effect of CAI on RIP2-NF-κB signaling in PBMCs from patients with BS. It was found that CAI did not affect the activation of RIP2, but significantly attenuated downstream events, including the phosphorylation of IKK and the nuclear translocation of p65, both in unstimulated BS PBMCs and in those stimulated by MDP combined with LPS. In agreement with our results, thalidomide has been reported to improve clinical symptoms in two BS patients by down-regulating NF-κB signaling [34]. Given that NF-κB activation drives the release of pro-inflammatory cytokines, and the elevated serum cytokines levels were documented in BS patients [35], we next examined the effect of CAI on cytokines secretion. Our results demonstrated that CAI treatment suppressed both the basal secretion and the MDP-induced increase of TNF-α and IL-6 in PBMCs from most BS patients. Furthermore, CAI also lowered the elevated levels of TNF-α, IL-1β, and IL-6 in response to MDP combinated with LPS. We noted that the responsiveness of PBMCs from different BS patients to CAI was variable. This inter-patient variability may be attributed to factors such as differences in disease duration, disease severity, and distinct NOD2 mutation sites. Future studies with larger sample sizes will help to better elucidate the effect of CAI. Together, these findings reveal that CAI inhibits the NF-κB pathway and subsequent pro-inflammatory cytokine release in BS PBMCs.

Based on the above valuable ex vivo findings, we further evaluated the therapeutic potential of CAI for BS in cellular models. Our previous studies have established that MDP-stimulated RAW264.7 cells serve as a fine cellular model of BS [28]. In this model, CAI treatment markedly inhibited the MDP-induced activation of both NF-κB and MAPK pathways, as well as the production of TNF-α, IL-1β, IL-6, and IL-8 triggered by MDP alone or in combination with LPS. This anti-inflammatory activity was also confirmed in iBMDM and THP-1 cell models. Notably, ENT, a soluble TNF receptor fusion protein used as a positive control, inhibited MDP-induced activation of NF-κB and MAPKs. This inhibition can be attributed to the blockade of autocrine/paracrine inflammatory amplification loops: ENT binds to TNF-α produced upon MDP stimulation, thereby preventing TNF-α from engaging TNFR1 and blocking the subsequent TNFR1-mediated amplification of NF-κB and MAPK signaling. This observation aligns with the known mechanism of ENT and validates the involvement of TNF-α-dependent amplification loops in our MDP-stimulated model. We next investigated whether CAI could target the core pathogenic event of BS—the constitutive NF-κB activation driven by NOD2 mutations. Compared to cells expressing NOD2-WT, HEK293T cells expressing the prevalent BS-associated mutants R334W and R334Q exhibited constitutive NF-κB activation, consistent with previous reports [11, 32, 33]. Importantly, CAI treatment potently suppressed this mutant-driven signaling. Furthermore, while MDP stimulation further enhanced NF-κB activity in cells expressing either WT or mutant NOD2, CAI significantly inhibited this induced activity. We noted that CAI inhibited NF-κB activity in NOD2-overexpressing HEK293T cells (Fig. 4) to a lesser extent than it suppressed cytokine secretion in BS patient PBMCs (Fig. 2) and RAW264.7 cells (Fig. 3). This discrepancy may be explained by two factors. First, the cellular contexts differ: HEK293T cells are non-immune cells with transient overexpression of NOD2, whereas PBMCs and macrophages are immune cells endogenously expressing NOD2 with intact and integrated inflammatory signaling networks. Second, cytokine production is controlled by a network of signaling pathways, including not only NF-κB but also MAPKs. Therefore, the pronounced inhibitory effect of CAI on inflammatory cytokine production likely reflects coordinated suppression of multiple pro-inflammatory pathways, rather than modulation of NF-κB alone. Nevertheless, the results demonstrate that CAI effectively counteracts NF-κB activation driven by pathogenic NOD2 mutations.

We next investigated the efficacy of CAI in animal models of BS. A mouse systemic inflammatory model of BS was established that displayed the elevated serum levels of TNF-α, IL-1β, IL-6, and IL-8; these cytokines were significantly reduced by CAI administration. The most relevant morbidity of BS is eye involvement, with uveitis being the most common manifestation, affecting 60% to 80% of BS patients [3, 10, 36–39]. Although uveitis typically emerges later than dermatitis and arthritis in the classic BS triad, it is often difficult to manage. Approximately 30% of patients with uveitis may progress to moderate-to-severe visual impairment [10, 36–39]. Given this clinical burden, we evaluated the effect of CAI on BS-associated uveitis in our established rat model induced by intravitreal MDP [29]. The administration of CAI alleviated the histopathological damage of uveitis, which was associated with suppressed activation of NF-κB and MAPKs pathways and reduced expression of pro-inflammatory cytokines in ocular tissues.

However, several limitations should be acknowledged. First, while this study focused on evaluating the effect of CAI on inflammatory responses in PBMCs from BS patients, the inclusion of PBMCs from healthy donors as controls would have further strengthened the findings. Second, due to limited BS patient sample availability, we were unable to perform a time-course experiment to capture the full kinetic profile of CAI’s effect on NF-κB activation in BS PBMCs. Although the 45 min time point was selected based on literature evidence [40], a time-course analysis would provide a more comprehensive understanding of the inhibitory dynamics. Third, the use of isolated monocytes might yield more convincing results; however, this requires a substantially larger blood volume. Given the rarity of BS and sample collection constraints, we used PBMCs in this study. Fourth, characterizing the composition of PBMC subpopulations in BS patients remains to be further investigated. Finally, in the experiments investigating the effect of CAI on p65 nuclear translocation, confocal microscopy would have been advantageous for visualizing subcellular localization with higher spatial resolution.

## Conclusions

In summary, we have shown that CAI potently inhibits the NF-κB and MAPK pathways downstream of NOD2, thereby suppressing the production of key pro-inflammatory cytokines in patient-derived PBMCs and various BS cellular models. We also validated this efficacy in vivo, where CAI ameliorated systemic inflammation and, most importantly, alleviated BS-associated uveitis by mitigating histopathological damage and inhibiting inflammatory signaling within ocular tissues. Collectively, these findings provide a basis for the development of CAI as a new potential drug candidate for the treatment of BS.

## Supplementary Information

Supplementary Material 1.

Supplementary Material 2.

## Data Availability

All data generated or analyzed during this study are included in this published article and further datasets are available from the corresponding author on reasonable request.

## Abbreviations

BS: Blau syndrome
CAI: Carboxyamidotriazole
CARDs: Caspase activation and recruitment domains
DMEM: Dulbecco’s modified Eagle’s medium
DMSO: Dimethyl sulfoxide
ELISA: Enzyme-linked immunosorbent assay
ETN: Etanercept
FBS: Fetal bovine serum
H&E: Hematoxylin and eosin
HRP: Horseradish peroxidase
iBMDM: Immortalized bone marrow-derived macrophage
IL: Interleukin
L18-MDP: MDP with a C18 fatty acid chain
LPS: Lipopolysaccharide
LRRs: Leucine-rich repeats
MAPKs: Mitogen-activated protein kinases
MDP: Muramyl dipeptide
NBDs: Nucleotide binding domains
NF-κB: Nuclear factor-κB
NLR: NOD-like receptor
NOD2: Nucleotide-binding oligomerization domain containing 2
PBMCs: Peripheral blood mononuclear cells
PEG 400: Polyethylene glycol 400
PMA: Phorbol 12-myristate 13-acetate
RIP2: Receptor interaction protein-2
SD: Sprague-Dawley
TNF: Tumor necrosis factor
WT: Wild type

## References

[1] Matsuda T, Kambe N, Ueki Y, Kanazawa N, Izawa K, Honda Y, et al. Clinical characteristics and treatment of 50 cases of Blau syndrome in Japan confirmed by genetic analysis of the NOD2 mutation. Ann Rheum Dis. 2020;79(11):1492–9. 32647028 10.1136/annrheumdis-2020-217320

[2] Iannuzzi MC, Rybicki BA, Teirstein AS, Sarcoidosis. N Engl J Med. 2007;357(21):2153–65. 18032765 10.1056/NEJMra071714

[3] Kaufman KP, Becker ML. Distinguishing Blau syndrome from systemic sarcoidosis. Curr Allergy Asthma Rep. 2021;21(2):10. 33560445 10.1007/s11882-021-00991-3 PMC9762981

[4] Miceli-Richard C, Lesage S, Rybojad M, Prieur AM, Manouvrier-Hanu S, Häfner R, et al. CARD15 mutations in Blau syndrome. Nat Genet. 2001;29(1):19–20. 11528384 10.1038/ng720

[5] Feerick CL, McKernan DP. Understanding the regulation of pattern recognition receptors in inflammatory diseases - a ‘Nod’ in the right direction. Immunology. 2017;150(3):237–47. 27706808 10.1111/imm.12677 PMC5290251

[6] Kim YG, Kamada N, Shaw MH, Warner N, Chen GY, Franchi L, et al. The Nod2 sensor promotes intestinal pathogen eradication via the chemokine CCL2-dependent recruitment of inflammatory monocytes. Immunity. 2011;34(5):769–80. 21565531 10.1016/j.immuni.2011.04.013 PMC3103637

[7] Pellegrini E, Desfosses A, Wallmann A, Schulze WM, Rehbein K, Mas P, et al. RIP2 filament formation is required for NOD2 dependent NF-κB signalling. Nat Commun. 2018;9(1):4043. 30279485 10.1038/s41467-018-06451-3 PMC6168553

[8] Wu D, Shen M. Two Chinese pedigrees of Blau syndrome with thirteen affected members. Clin Rheumatol. 2018;37(1):265–70. 28721627 10.1007/s10067-017-3758-7

[9] Rosé CD, Pans S, Casteels I, Anton J, Bader-Meunier B, Brissaud P, et al. Blau syndrome: cross-sectional data from a multicentre study of clinical, radiological and functional outcomes. Rheumatology (Oxford). 2015;54(6):1008–16. 25416713 10.1093/rheumatology/keu437

[10] Sfriso P, Caso F, Tognon S, Galozzi P, Gava A, Punzi L. Blau syndrome, clinical and genetic aspects. Autoimmun Rev. 2012;12(1):44–51. 22884558 10.1016/j.autrev.2012.07.028

[11] Kanazawa N, Okafuji I, Kambe N, Nishikomori R, Nakata-Hizume M, Nagai S, et al. Early-onset sarcoidosis and CARD15 mutations with constitutive nuclear factor-kappaB activation: common genetic etiology with Blau syndrome. Blood. 2005;105(3):1195–7. 15459013 10.1182/blood-2004-07-2972

[12] Nagakura T, Wakiguchi H, Kubota T, Yamatou T, Yamasaki Y, Nonaka Y, et al. Tumor necrosis factor inhibitors provide longterm clinical benefits in pediatric and young adult patients with Blau syndrome. J Rheumatol. 2017;44(4):536–8. 28604349 10.3899/jrheum.160672

[13] Lu L, Shen M, Jiang D, Li Y, Zheng X, Li Y, et al. Blau syndrome with good responses to tocilizumab: a case report and focused literature review. Semin Arthritis Rheum. 2018;47(5):727–31. 29110911 10.1016/j.semarthrit.2017.09.010

[14] Lassoued Ferjani H, Kharrat L, Ben Nessib D, Kaffel D, Maatallah K, Hamdi W. Management of Blau syndrome: review and proposal of a treatment algorithm. Eur J Pediatr. 2024;183(1):1–7. 37735224 10.1007/s00431-023-05204-9

[15] Kohn EC, Felder CC, Jacobs W, Holmes KA, Day A, Freer R, et al. Structure-function analysis of signal and growth inhibition by carboxyamido-triazole, CAI. Cancer Res. 1994;54(4):935–42. 8313384

[16] Moody TW, Chiles J, Moody E, Sieczkiewicz GJ, Kohn EC. CAI inhibits the growth of small cell lung cancer cells. Lung Cancer. 2003;39(3):279–88. 12609566 10.1016/s0169-5002(02)00525-1

[17] He Y, Zhu L. Inhibitory effect of carboxyamidotriazole on the proliferation of human eosinophil EOL-1. Herald Med. 2024;43(6):911–5.

[18] Zhu L, Li J, Guo L, Yu X, Wu D, Luo L, et al. Activation of NALP1 inflammasomes in rats with adjuvant arthritis; a novel therapeutic target of carboxyamidotriazole in a model of rheumatoid arthritis. Br J Pharmacol. 2015;172(13):3446–59. 25799914 10.1111/bph.13138 PMC4500378

[19] Du X, Chen W, Wang Y, Chen C, Guo L, Ju R, et al. Therapeutic efficacy of carboxyamidotriazole on 2,4,6-trinitrobenzene sulfonic acid-induced colitis model is associated with the inhibition of NLRP3 inflammasome and NF-κB activation. Int Immunopharmacol. 2017;45:16–25. 28152446 10.1016/j.intimp.2017.01.015

[20] Zhou Y, Yang X, Liu J, Yang M, Ye C, Zhu L. Carboxyamidotriazole alleviates pannus formation and cartilage erosion in rats with adjuvant arthritis. Heliyon. 2023;9(9):e20105. 37809969 10.1016/j.heliyon.2023.e20105 PMC10559848

[21] Duan M, Shen M, Zhou Y, He Y, Guo Z, Ye C, et al. AIM2 and NLRC4-driven inflammasome activation in adult-onset Still’s disease and the preliminary therapeutic effect exploration of carboxyamidotriazole. Clin Rheumatol. 2023;42(6):1635–43. 36418508 10.1007/s10067-022-06443-1

[22] Duan M, Shen M, He Y, Ye C, Zhu L. Therapeutic effect of carboxyamidotriazole on IL-1β-mediated autoinflammatory dseases. Herald Med. 2023;42(10):1502–7.

[23] Liu J, Yang M, Zhu L. Carboxyamidotriazole ameliorates experimental psoriasis via downregulating the expressions of cytokines and antimicrobial peptide S100A7. Acta Pharm Sinica. 2024;59(11):3085–93.

[24] Yang M, Lu S, Li J, Zhu L, Carboxyaminotriazole. A bone savior in collagen-induced arthritis-Halting osteoclastogenesis via interleukin-1β downregulation. Life Sci. 2025;364:123440. 39920985 10.1016/j.lfs.2025.123440

[25] Zhang X, Liu J, Yang M, Li J, Zhu L. Carboxyamidotriazole regulates the function of salivary gland epithelial cells and B cells to alleviate experimental Sjögren’s disease in mice. Int J Med Sci. 2025;22(10):2362–72. 40386058 10.7150/ijms.111897 PMC12080577

[26] Lu S, Duan M, Guo Z, Zhou Y, Wu D, Zhang X, et al. Carboxyamidotriazole exerts anti-inflammatory activity in lipopolysaccharide-induced RAW264.7 macrophages by inhibiting NF-κB and MAPKs pathways. Exp Ther Med. 2020;20(2):1455–66. 32742379 10.3892/etm.2020.8889 PMC7388320

[27] Chen J, Luo Y, Zhao M, Wu D, Yang Y, Zhang W, et al. Effective treatment of TNFα inhibitors in Chinese patients with Blau syndrome. Arthritis Res Ther. 2019;21(1):236. 31718710 10.1186/s13075-019-2017-5 PMC6852754

[28] Song H, Ye C, Zhu L. Establishment of cell models for Blau syndrome. Basic Clin Med. 2022;42(3):406–10.

[29] Song H, Yang X, Ye C, Zhu L. Establishment of animal model of Blau syndrome. Chin Pharmacol Bull. 2023;39(6):1195–9.

[30] Agarwal RK, Silver PB, Caspi RR. Rodent models of experimental autoimmune uveitis. Methods Mol Biol. 2012;900:443–69. 22933083 10.1007/978-1-60761-720-4_22 PMC3810964

[31] Yang X, Ye C, Zhu L. Research progress of NOD2-mediated signaling pathways and relationship with autoinflammatory diseases and its inhibitors. Acta Pharm Sinica. 2023;58(4):899–908.

[32] Ebrahimiadib N, Samra KA, Domina AM, Stiles ER, Ewer R, Bocian CP, et al. A Novel NOD2-associated mutation and variant Blau syndrome: phenotype and molecular analysis. Ocul Immunol Inflamm. 2018;26(1):57–64. 27419275 10.1080/09273948.2016.1185529

[33] Chamaillard M, Philpott D, Girardin SE, Zouali H, Lesage S, Chareyre F, et al. Gene-environment interaction modulated by allelic heterogeneity in inflammatory diseases. Proc Natl Acad Sci U S A. 2003;100(6):3455–60. 12626759 10.1073/pnas.0530276100 PMC152314

[34] Yasui K, Yashiro M, Tsuge M, Manki A, Takemoto K, Yamamoto M, et al. Thalidomide dramatically improves the symptoms of early-onset sarcoidosis/Blau syndrome: its possible action and mechanism. Arthritis Rheum. 2010;62(1):250–7. 20039400 10.1002/art.25035

[35] Aróstegui JI, Arnal C, Merino R, Modesto C, Antonia Carballo M, Moreno P, et al. NOD2 gene-associated pediatric granulomatous arthritis: clinical diversity, novel and recurrent mutations, and evidence of clinical improvement with interleukin-1 blockade in a Spanish cohort. Arthritis Rheum. 2007;56(11):3805–13. 17968944 10.1002/art.22966

[36] Kurokawa T, Kikuchi T, Ohta K, Imai H, Yoshimura N. Ocular manifestations in Blau syndrome associated with a CARD15/Nod2 mutation. Ophthalmology. 2003;110(10):2040–4. 14522785 10.1016/S0161-6420(03)00717-6

[37] Suresh S, Tsui E. Ocular manifestations of Blau syndrome. Curr Opin Ophthalmol. 2020;31(6):532–7. 33009086 10.1097/ICU.0000000000000705

[38] Maccora I, Wouters C, Rosè CD, Maniscalco V, de Masi S, Mastrolia MV, et al. Treatment of uveitis in Blau syndrome: a systematic review and meta-analysis. J Autoimmun. 2025;153:103401. 40147219 10.1016/j.jaut.2025.103401

[39] Chinese Society of Dermatology, China Dermatologist Association. Expert consensus on diagnosis and treatment of Blau syndrome (2024). Chin J Dermatology. 2024;57(11):1004–10.

[40] Yang Y, Yin C, Pandey A, Abbott D, Sassettic C, Kelliher MA. NOD2 pathway activation by MDP or Mycobacterium tuberculosis infection involves the stable polyubiquitination of Rip2. J Biol Chem. 2007;282(50):36223–9. 17947236 10.1074/jbc.M703079200

