# Supplementary figures and images for: Inactivation of NF-κB and MAPKs confers the potent therapeutic effect of carboxyamidotriazole on Blau syndrome
Source: Arthritis Res Ther. 2026 Mar 26;28:105. doi: 10.1186/s13075-026-03800-2 (PMC13141424; doi:10.1186/s13075-026-03800-2)

**Fig. 1A**

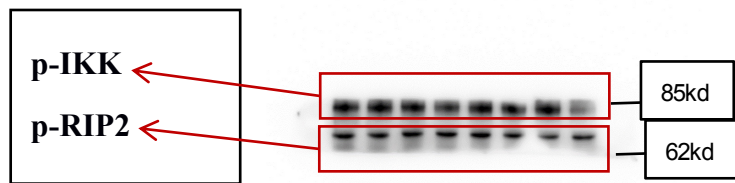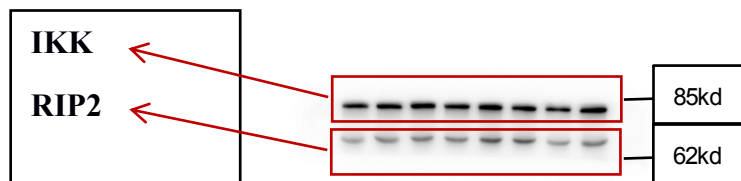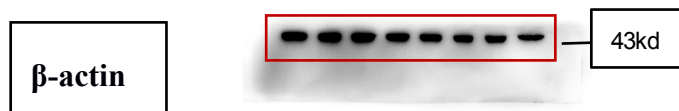

**Fig. 3A**

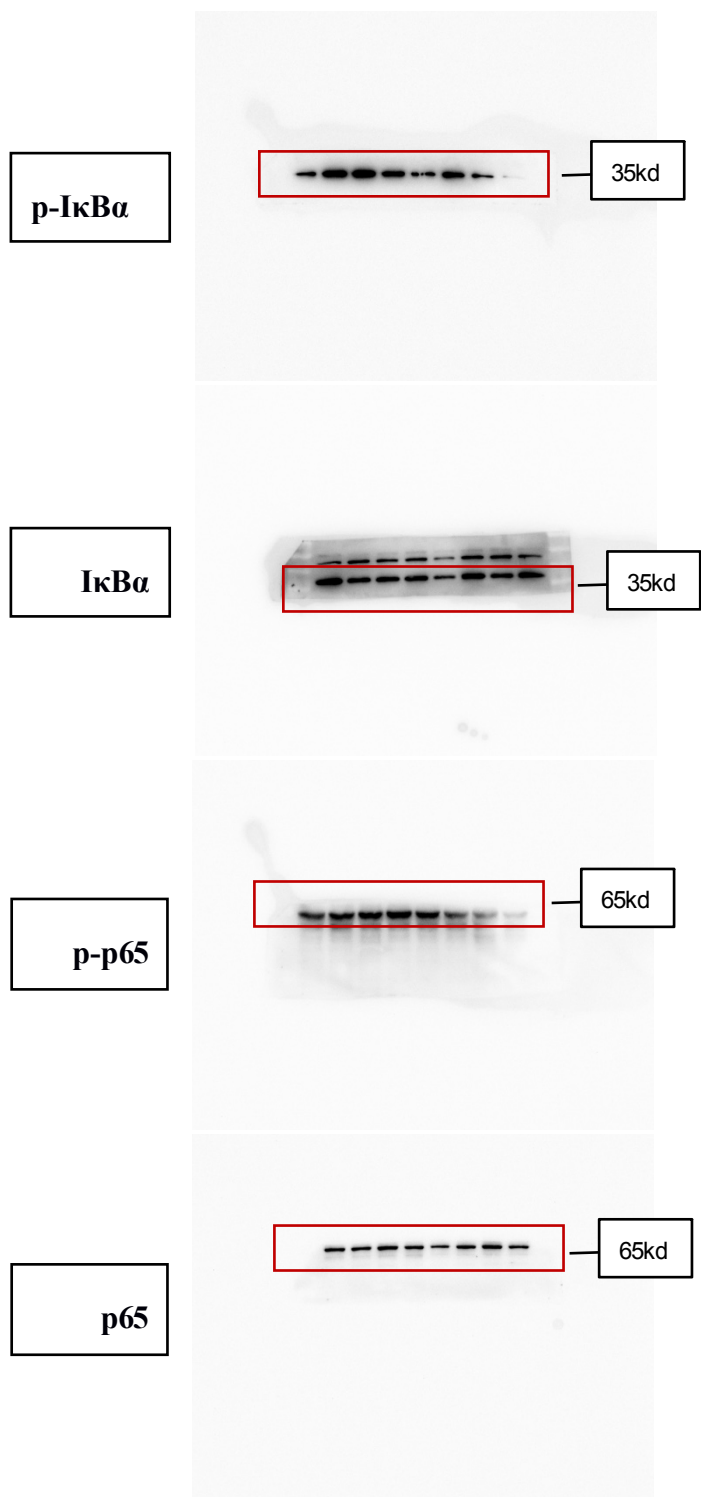

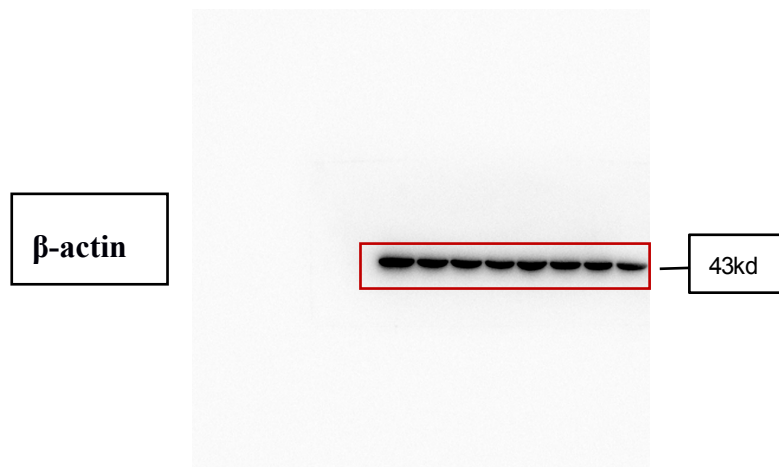

**Fig. 3B**

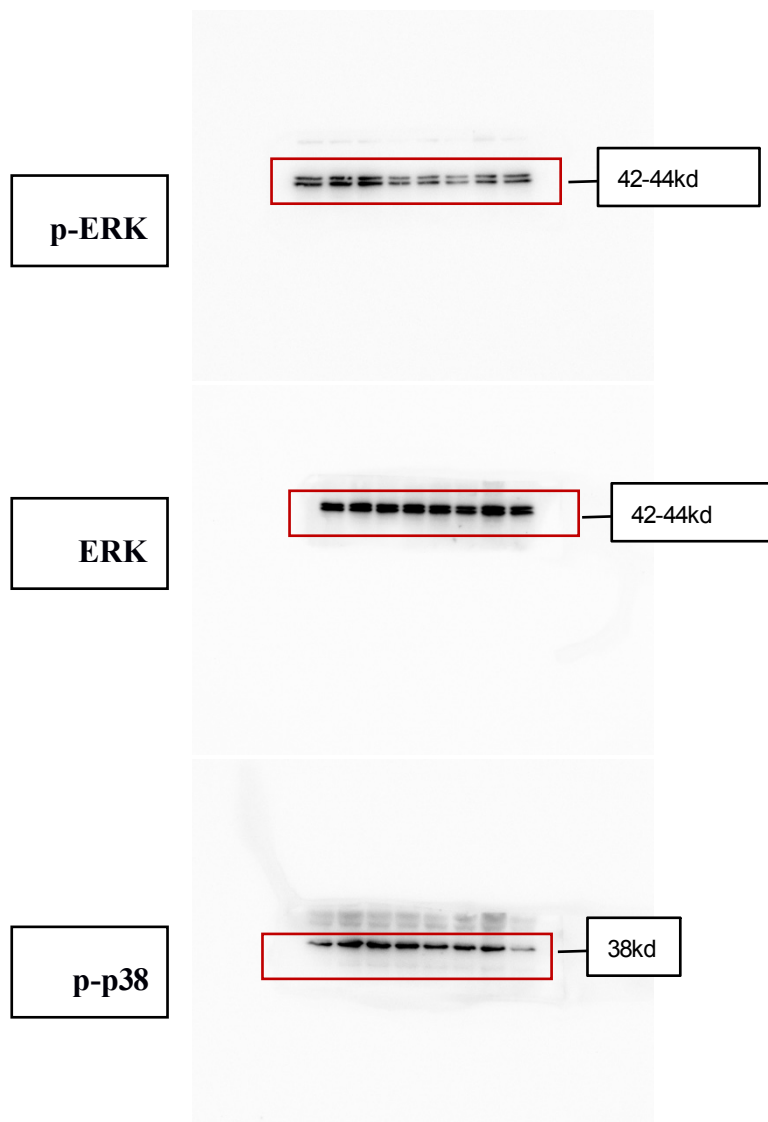

**p38**

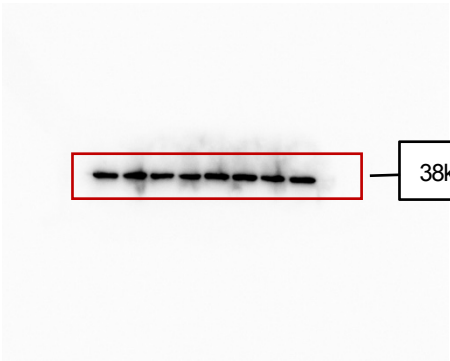

38kd

**p-JNK**

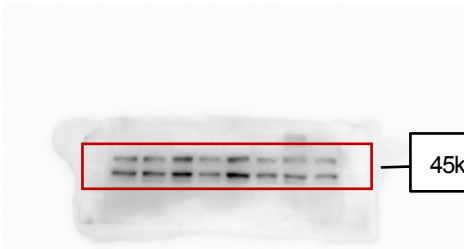

45kd

**JNK**

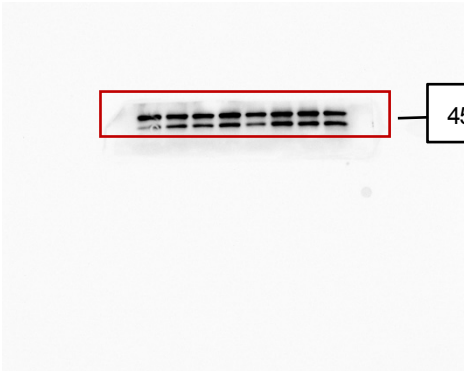

45kd

**$\beta$ -actin**

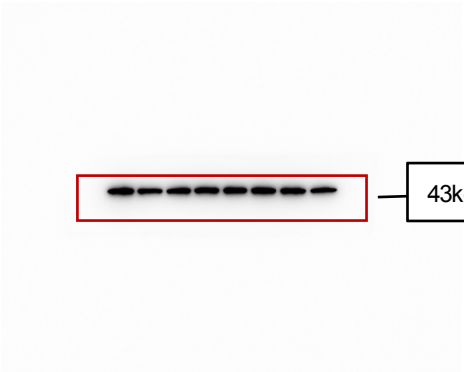

43kd

Supplement: Supplementary file 1 — Supplementary Material 1. [file 13075_2026_3800_MOESM1_ESM.pdf]
