# Supplementary material for: Inactivation of NF-κB and MAPKs confers the potent therapeutic effect of carboxyamidotriazole on Blau syndrome
Source: Arthritis Res Ther. 2026 Mar 26;28:105. doi: 10.1186/s13075-026-03800-2 (PMC13141424; doi:10.1186/s13075-026-03800-2)
Supplement: Supplementary file 2 — Supplementary Material 2. [file 13075_2026_3800_MOESM2_ESM.docx]

# Supplemental information

# Inactivation of NF-κB and MAPKs confers the potent therapeutic effect of carboxyamidotriazole on Blau syndrome

Haoxin Song^1 †^, MS; Yang Li^1 †^, Ms; Yuxin Wang^1 †^, Ms; Mengyuan Duan^1 †^, PhD; Na Wu^2^, MD; Yu Du^1^, Ms; Ru Xu^1^, Ms; Quanlin Chen^1^, Ms; Min Shen^2^ *, MD; Fang Wei^3^ *, MD; Lei Zhu^1 3 4*^, MD

† These authors contributed equally to this work

^1^ Department of Pharmacology, Institute of Basic Medical Sciences, Chinese Academy of Medical Sciences and School of Basic Medicine, Peking Union Medical College, Beijing 100005, China

^2^ Department of Rare Diseases, Peking Union Medical College Hospital (PUMCH), Chinese Academy of Medical Sciences & Peking Union Medical College; State Key Laboratory of Complex Severe and Rare Diseases, PUMCH; Department of Rheumatology and Clinical Immunology, PUMCH; National Clinical Research Center for Dermatologic and Immunologic Diseases (NCRC-DID), Ministry of Science & Technology; Key Laboratory of Rheumatology and Clinical Immunology, Ministry of Education, Beijing 100730, China

^3^ School of Pharmacy, Bengbu Medical University, Bengbu 233030, China

^4^ Medical Epigenetics Research Center, Chinese Academy of Medical Sciences, Beijing 100005, China

**Corresponding author:**

Lei Zhu, [leizhu2004@126.com](mailto:leizhu2004@126.com)

Min Shen, [shenmpumch@163.com](mailto:shenmpumch@163.com)

Fang Wei, [weifangmailbox@126.com](mailto:weifangmailbox@126.com)

**Supplementary Table**

**Table S1 Demographics of study subjects**

| Variable | Patient 1 | Patient 2 | Patient 3 | Patient 4 | Patient 5 |
| --- | --- | --- | --- | --- | --- |
| Age (years old) | 32 | 8 | 36 | 30 | 25 |
| Gender | female | male | female | male | female |
| Disease course (years) | 26 | 6 | 30 | 29 | 19 |
| *NOD2* variants | R334W heterozygous | R334W heterozygous | R334W heterozygous | R471C/H496P compound heterozygous | R334Q heterozygous |

**Supplemental Figures**


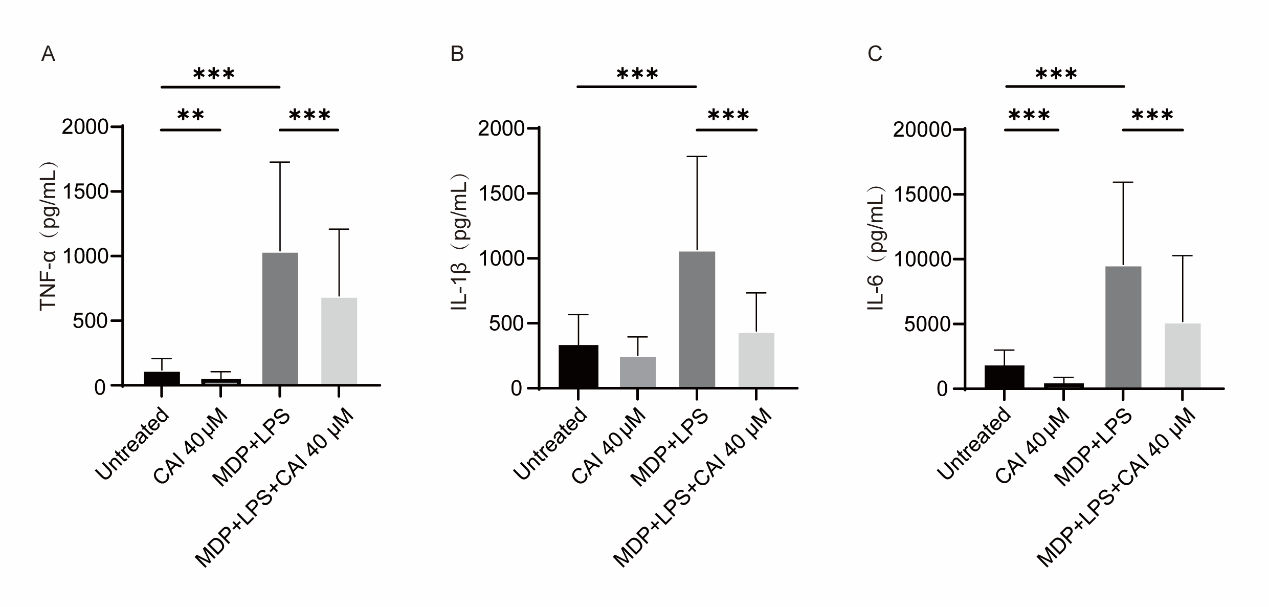


**Fig. S1** **Pooled analysis of the four groups common to all five patients.** Data from the four experimental conditions (untreated, CAI 40 μM, MDP+LPS, and MDP+LPS+ CAI 40 μM) for all five patients in Fig. 2 were pooled and analyzed collectively. The values are mean±SD; for each patient, n=2-5 per experimental condition. ***p*<0.01 and ****p<*0.001.


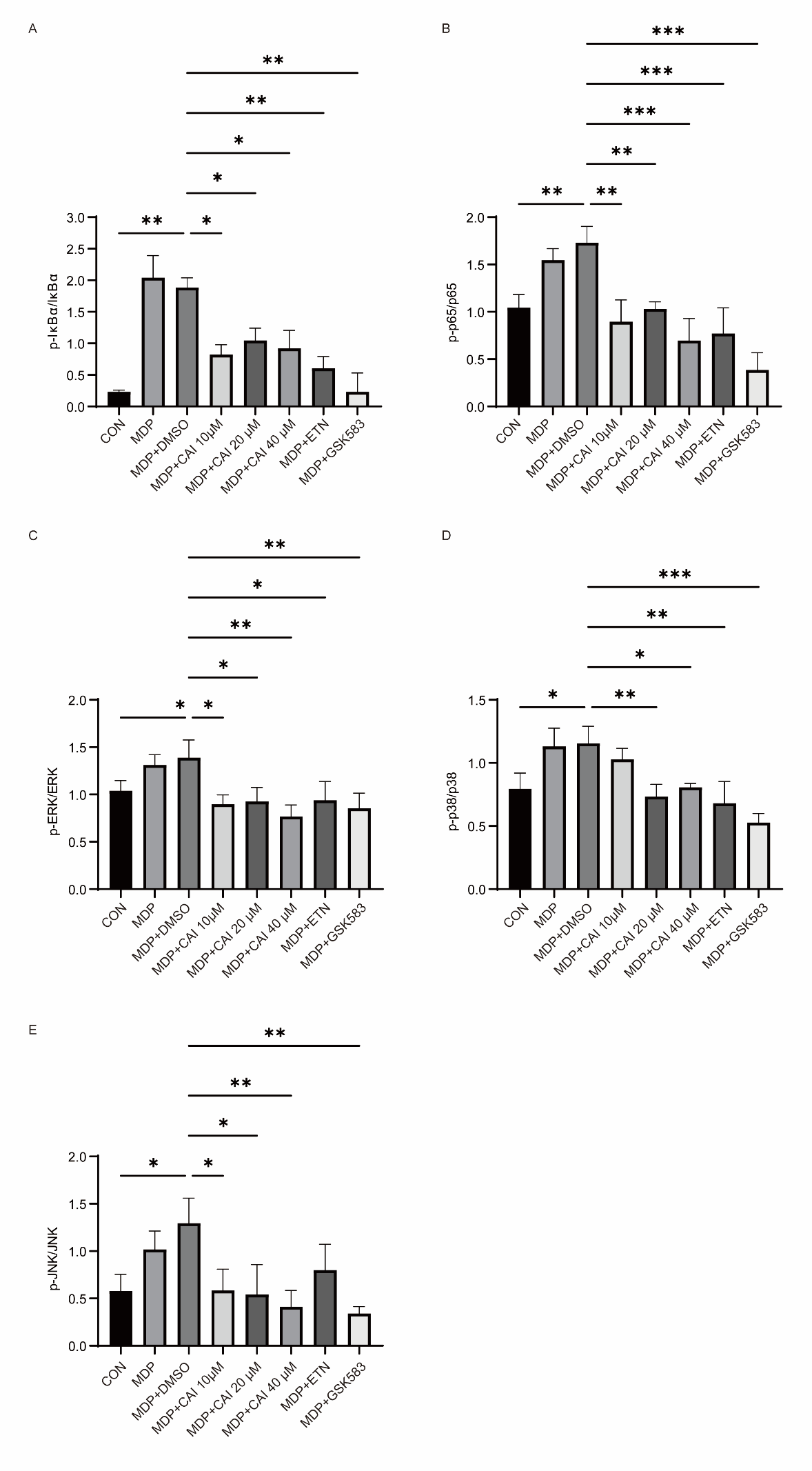


**Fig. S2 Quantification of immunoblot bands in Fig. 3A and B.** The ratios of phosphorylated to total proteins were calculated following densitometric analysis. The values are mean±SD, n=3 per group. **p*<0.05, ***p*<0.01 and ****p*<0.001.


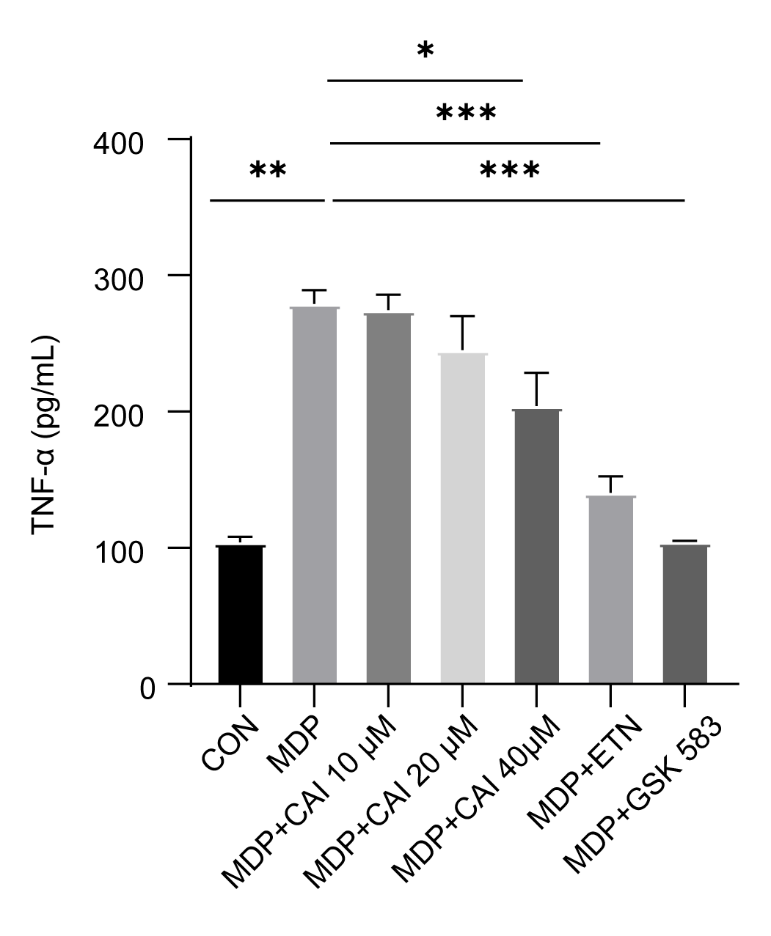


**Fig. S3 Effect of CAI on TNF-α secretion by MDP-induced iBMDM** **model.** iBMDMs were stimulated with 10 μg/mL MDP for 22 h, either alone or in combination with CAI or with the positive control drugs (ETN and GSK583). TNF-α level in the supernatants were measured by ELISA. The values are mean±SD, n=3 per group. **p*<0.05, ***p*<0.01 and ****p<*0.001.


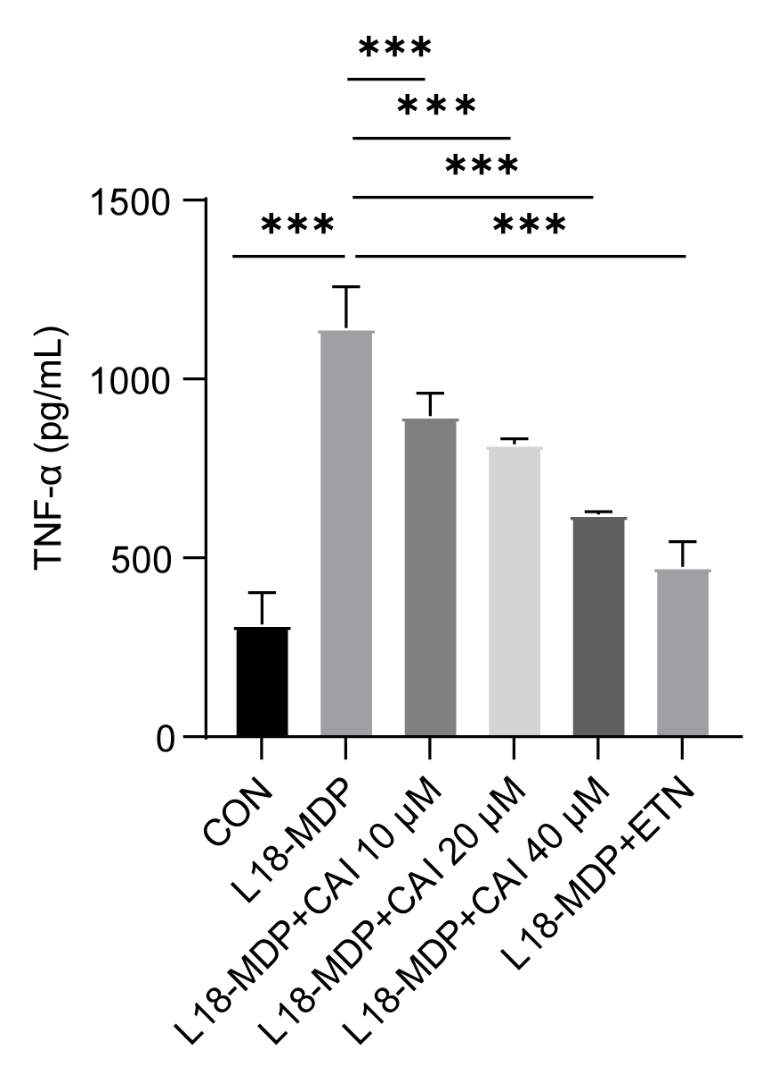


**Fig. S4 Effect of CAI on TNF-α secretion by L18-MDP-induced THP-1 cell model.** PMA differentiated THP-1 cells were stimulated with 0.2 μg/mL L18-MDP for 22 h, either alone or in combination with CAI or ETN. TNF-α level in the supernatants were measured by ELISA. The values are mean±SD, n=3 per group. ****p<*0.001.
